# Obliquity-paced climate change recorded in Antarctic debris-covered glaciers

**DOI:** 10.1038/ncomms14194

**Published:** 2017-02-10

**Authors:** Sean L. Mackay, David R. Marchant

**Affiliations:** 1 Department of Earth and Environment, Boston University, Boston, Massachusetts 02215, USA

## Abstract

The degree to which debris-covered glaciers record past environmental conditions is debated. Here we describe a novel palaeoclimate archive derived from the surface morphology and internal debris within cold-based debris-covered glaciers in Antarctica. Results show that subtle changes in mass balance impart major changes in the concentration of englacial debris and corresponding surface topography, and that over the past ∼220 ka, at least, the changes are related to obliquity-paced solar radiation, manifest as variations in total summer energy. Our findings emphasize solar radiation as a significant driver of mass balance changes in high-latitude mountain systems, and demonstrate that debris-covered glaciers are among the most sensitive recorders of obliquity-paced climate variability in interior Antarctica, in contrast to most other Antarctic archives that favour eccentricity-paced forcing over the same time period. Furthermore, our results open the possibility that similar-appearing debris-covered glaciers on Mars may likewise hold clues to environmental change.

## Abstract (web summary)

The degree to which debris-covered glaciers record past environmental conditions is debated. Here, the authors show that obliquity-paced variations in solar radiation over the past ∼220 ka are expressed in Mullins glacier as repetitive changes in englacial debris and corresponding surface topography.

Glacial cycles during the mid-Pleistocene transition (700–1,250 ka) underwent a major reorganization in periodicity, from ∼41 to ∼100 ka, followed later by a second transition at ∼400 ka, when the amplitude of interglacial thermal extremes increased (the mid-Brunhes event)^1^. These transitions are of particular interest because they established, and then reinforced, a ∼100 ka periodicity^2^,^3^,^4^,^5^,^6^ that is seemingly at odds with the pacing of known variations in solar forcing^7^ arising from changes in Earth's obliquity (paced at ∼41 ka) and precession (paced at ∼21 ka). Although several models have been proposed to explain the onset and maintenance of the ∼100 ka world^8^,^9^, we ask whether some high-latitude glacial systems in Antarctica still maintain a heightened sensitivity to obliquity forcing—even as the pace of global glacial cycles slowed.

To address this question, we analysed the surface and internal characteristics of two small (<6.5 km long and ∼125 m thick), north-facing debris-covered alpine glaciers^10^,^11^,^12^ in the southern Transantarctic Mountains, the Mullins (77.89°S, 160.58°W, 1,557 m MSL) and Friedman (77.90°S, 160.51°W, 1,540 m MSL) glaciers (Fig. 1). These glaciers are among the oldest reported alpine glaciers in Antarctica^11^,^13^,^14^ and have the potential to register climate change on timescales of 10^5^–10^6^ a. Using a numerical model of glacial mass balance, we explore the sensitivity of these glaciers to the range of climate forcing experienced in the region over the past ∼250 ka. These results are combined with morphological observations, chronological constraints and a geomorphic model for debris entrainment to detect tractable links between past climate change and preserved glacier structure.

## Results

### Glaciology and critical zone dynamics

Located at similar elevations and <∼2 km apart, Mullins and Friedman glaciers share identical hyper-arid polar-desert climate conditions, including summer average temperatures and relative humidity in their ice ablation zones (−14 °C and 53%, respectively^12^ (Supplementary Fig. 1, see ‘Methods' section)). Modern precipitation rates (including atmospheric deposition and wind-transported snow) are poorly constrained over these glaciers, but likely fall within the range of ∼4–10 cm a^−1^ (ref. 15) (see ‘Methods' section, Supplementary Fig. 1). Each glacier includes a small ice-accumulation zone, dotted in places with isolated gravel-, cobble-, and boulder-sized clasts, that grades down valley into an increasingly thick (0.1–0.5 m) layer of surface debris that mantles buried glacier ice in the ablation zone with sharp, dry contacts. The debris is sourced exclusively from rockfall off bedrock cliffs at valley headwalls^10^,^12^; rocks that fall on ice-accumulation zones are subsequently buried and transported down valley—as englacial debris—before rising upward toward the ice surface as overlying ice sublimes; rocks that land within ice-ablation zones travel only as supraglacial debris. Both glaciers are cold-based and frozen to their bed^12^; all down valley ice flow is accommodated by internal deformation, rather than by basal slip, and basal entrainment is either completely absent^12^ or limited to a negligible amount of fine-grained material near the base and terminus of the glacier^16^,^17^.

**Figure 1:**
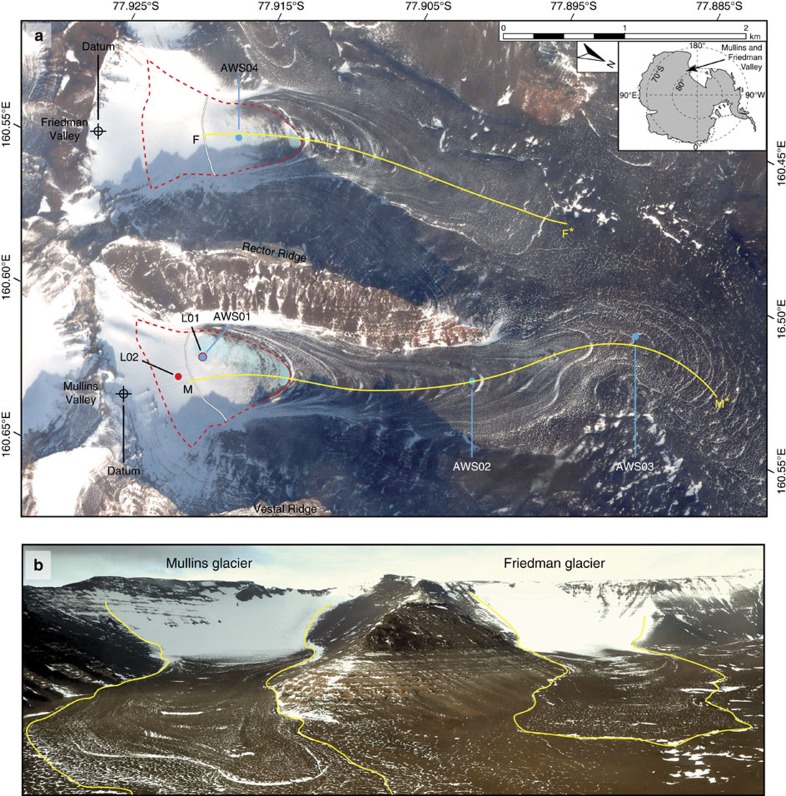
Mullins and Friedman debris-covered glaciers. (**a**) GeoEye01 (1.5 m resolution, January 2004) satellite image of Mullins and Friedman glaciers. Automatic weather stations (AWS 1–4) and model locations/data points (L01 and L02) (see 'Methods' section) are marked. The critical zone for both glaciers is outlined with a red dashed line. The grey lines within the critical zones mark the inferred approximate equilibrium lines. The yellow line near the central flow line of each glacier marks the location of the GPR transect used for the subsurface interpretation shown in Fig. 2b. (**b**) Oblique aerial photograph looking SSE up Beacon Valley, Antarctica. The approximate extents of Mullins and Freidman debris-covered glaciers are outlined in yellow.

The antiquity of debris-covered alpine glaciers in polar deserts is due largely to a dynamic balance between (1) mass wasting and snow accumulation at headwall cliffs and (2) exceedingly slow rates of ice loss beneath protective supraglacial debris^18^. These conditions impart unusual ice dynamics that critically differentiate debris-covered alpine glaciers from typical (non-debris-covered) glaciers. Namely, ablation rates in debris-covered glaciers are highest near glacier heads, rather than at low-elevation glacial termini^10^; as a result, glacier sensitivity to environmental change is greatest in ice-accumulation and uppermost ablation zones, which lack protective overburden^19^,^20^,^21^. Given this unusual scenario, we refer to the spatial area incorporating the ice-accumulation and uppermost ablation zones on debris-covered glaciers as the glacier critical zone (Fig. 1).

**Figure 2:**
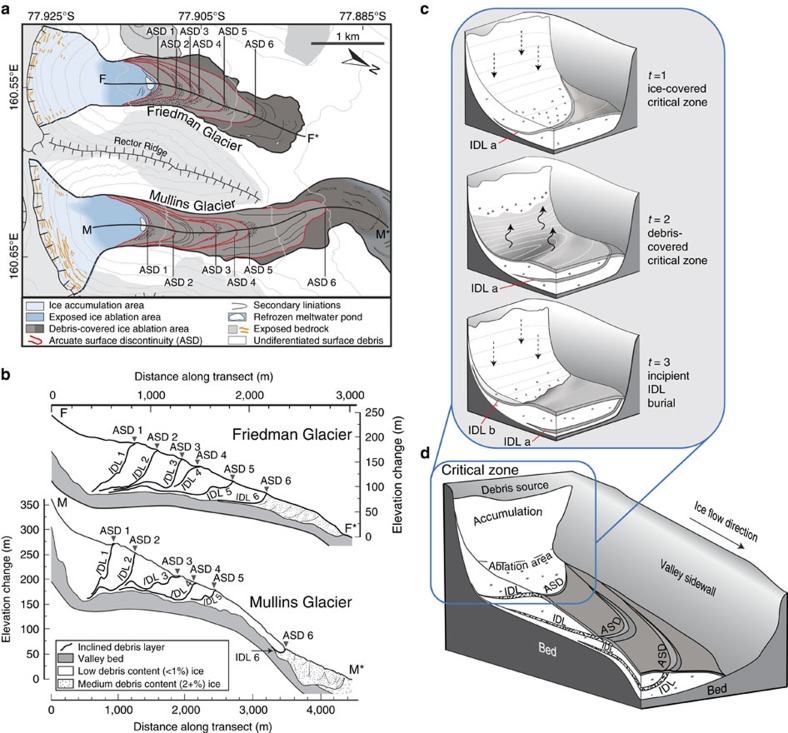
The morphology development of inclined debris layer. (**a**) Mapped surface features in Mullins and Friedman valleys showing the locations of six ASD (see text) for each glacier. (**b**) Subsurface structure of Mullins and Friedman glaciers as interpreted from ground-penetrating radar and ice core data^12^(see Supplementary Figs 2,3); six IDL are present in each glacier. (**a**,**b**) are modified from ref. 12. (**c**) Conceptual model for the formation of ASD/IDL in the critical zone. During conditions favourable for net ice accumulation (*t*=1), the glacier maintains exposed ice in the upper ablation area and surface debris gradually thickens down valley. When conditions shift to net ablation (*t*=2), significant ice loss occurs near the valley headwall (unprotected ice, glacier critical zone, see text), but minimal ice loss occurs beneath thick debris down valley. As ice loss progresses, a spoon-shaped hollow may form near the headwall that is ultimately capped by a lag of debris; this capping debris develops through the continued emplacement of rocks via rockfall and from the accumulation of scattered englacial debris that is brought to the ice surface as overlying ice sublimes. When the conditions shift to net ice accumulation (*t*=3), the rocky surface lag and spoon-shaped hollow are in-filled and buried with snow and glacier ice (even with horizontal ice flow); this buried rocky layer becomes an IDL that deforms and moves down valley with glacier flow. (**d**) Upon multiple repetitions, a series of IDL are preserved marking successive climate transitions. Each IDL manifests at the glacier surface as an ASD.

At present, exposed ice in the critical zone of both Mullins and Friedman glaciers ablates at ∼7 cm a^−1^. Down glacier, the rate of ice loss decreases rapidly and ranges from a maximum of ∼0.05 cm a^−1^ (at the onset of continuous surface debris cover <5 cm thick) to ∼6.6 × 10^−7^ cm a^−1^ beneath debris ∼50 cm in thickness^10^. Without the influx of ice flow from high-elevation areas in ice-accumulation zones, enhanced ice loss in critical zones would dramatically lower ice-surface elevations (relative to down valley ice protected by thick debris cover) and result in the development of local spoon-shaped depressions^19^,^21^ in the glacier critical zone that terminate down valley at the margin of thick, debris-covered glacier ice (Fig. 2); such spoon-shaped hollows at the head of debris-covered glaciers are found in nearby valleys in Antarctica^18^, and even on Mars^22^,^23^.

### Inclined debris layers in Mullins and Friedman glaciers

Twenty-four kilometres of overlapping and multiple-frequency ground-penetrating radar (GPR) transects, 21 shallow ice cores, and >40 near-surface ice excavations on Mullins and Friedman glaciers were collected and analysed by Mackay, Marchant^12^; the data reveal a near-identical pattern of six inclined bands of concentrated rocks and sediment in Mullins and Friedman glaciers (Inclined Debris Layers, IDLs, ∼10–35% debris by volume) separated by clean glacier ice (<∼1% debris by volume)^12^ (Fig. 1, Supplementary Figs 2,3). Each IDL in Mullins and Friedman glaciers rests several metres above their respective glacier beds and slopes upward to intersect buried ice surfaces at corresponding arcuate topographic ridges (arcuate surface discontinuities, ASD)^12^ (Figs 1 and 2; Supplementary Figs 2,3).

Following^12^,^24^, and more broadly^19^,^25^,^26^ these IDL are thought to reflect recurrent environmental change in glacier critical zones. Expanding on this proposition, Mackay, Marchant^12^ argued that observed patterns of clean glacier ice interspersed with layers of englacial rocky debris most likely reflect alternating changes in net ice accumulation and ice ablation, with possible implications for rates of rockfall delivery to glacier critical zones (Fig. 2c,d). If correct, IDL originate during times of net ice ablation, when persistent low-magnitude rockfall and/or sublimation of ice containing scattered debris (∼1%) produce a surface lag of rocks and sediment in critical zones that ultimately slows the loss of subjacent ice. During renewed net ice accumulation, these rocky lags are buried by snow and ice, deform and flow englacially down valley (through plastic deformation of surrounding glacier ice, for example, refs 25, 27, 28), and intersect the surface of buried ice at ASDs. The similar spacing and geometry of IDL/ASD for two separate, cold-based glaciers with different bed configurations and valley morphometries^12^ implicates regional climate change as the fundamental driver in their formation^12^. Specifically, the repetitive pattern of six IDL/ASD on both Mullins and Friedman glaciers highlight six periods of negative mass balance (increased ablation and/or decreased accumulation) separated by five periods of increased mass balance (decreased ablation and/or increased accumulation).

### Sensitivity to climate forcing

**Figure 3:**
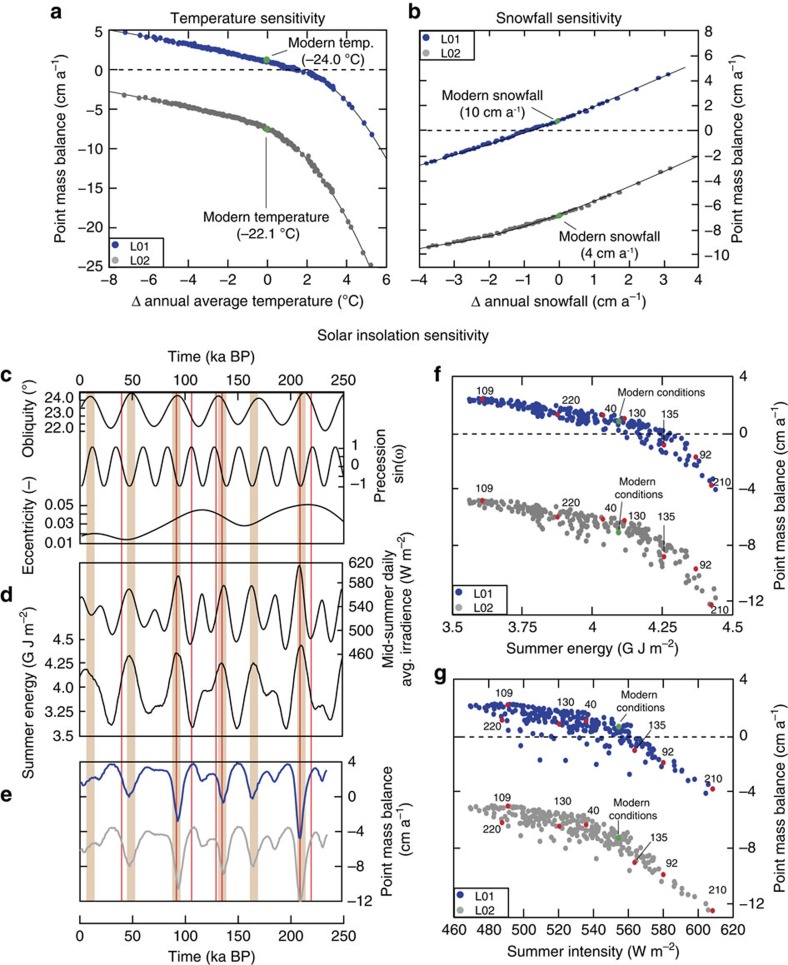
Mass balance in the critical zone of Mullins Glacier as a function of single parameter changes in climate forcing. Results are displayed for baseline environmental conditions at two sites (L01, 1,642 m elevation; L02 1,542 m elevation) (Fig. 1). (**a**) Mass balance sensitivity to changes in average annual air temperature, over the magnitude of temperature change recorded at Taylor Dome. The results show a non-linear response to warming air temperatures. (**b**) Sensitivity to changes in annual snowfall (with snow plotted as cm water equivalent). The slightly non-linear response is due to increased periods of relatively high albedo for elevated snow conditions. Bottom-left panels show changes in solar forcing functions over the last 250 ka and the associated mass balance response: (**c**) Orbital parameters; (**d**) December 21 top-of-atmosphere solar intensity (upper curve), and integrated summer energy (lower curve)^32^ computed for a threshold of 350 W m^−2^ resulting from the orbital configurations of **c**. The summer energy is defined as the sum of insolation intensity exceeding a given threshold^31^,^32^; (**e**) Modelled point mass balance over the 250 ka time period at L01 (grey line) and L02 (blue line) for the orbital configurations shown in **c**. Light tan, vertical bars in **c**,**d**,**e** mark peaks in summer energy and show that this quantity essentially changes in pace with obliquity variations^31^. Bottom-right panels show point mass balance plotted as a function of summer energy (**f**) and mid-summer solar intensity (**g**). Small red dots correspond to values for arbitrary time periods plotted as red vertical lines across **c**,**d**,**e**. Summer energy, which incorporates changes in summer duration as well as intensity, displays the strongest correlation with mass balance.

What process might drive similar changes in mass balance in the glacier critical zones of both Mullins and Friedman glaciers? To address this question, we use a numerical energy balance model (EBM) for point mass balance (accumulation – ablation) as input to our conceptual model for the formation of rocky surface lags in critical zones of debris-covered glaciers (Fig. 3, ‘Methods' section). The EBM, based on the distributed energy balance model (DEBAM) of Hock and Holmgren^29^ is first tuned to modern conditions and validated with high-temporal resolution micrometeorological measurements and local observational data (see ‘Methods' section). Next, individual components of the measured micrometeorological input data are adjusted systematically to test the modelled mass balance response to various forcing conditions. Through this modelling exercise, we evaluate the climate sensitivity of glacier critical zones and, by extension, the formation of IDL, under conditions of altered temperature, precipitation, solar intensity and summer duration. The results show that mass balance changes (positive verses negative) and resulting IDL production/cessation is possible with minor perturbations in atmospheric temperature, precipitation and/or solar forcing (Fig. 3).

### Obliquity-paced IDL formation in the late Pleistocene

**Figure 4:**
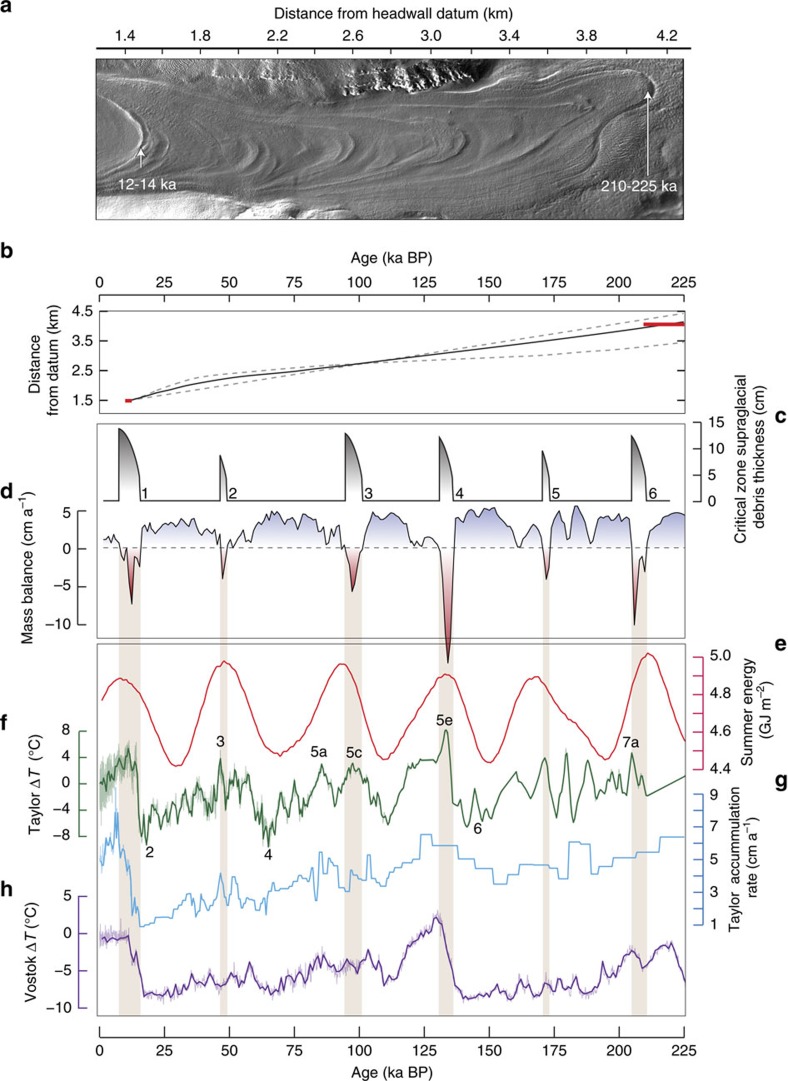
Correlations among changes in the surface morphology of Mullins Glacier and results from mass-balance models and climate forcing functions. (**a**) Hillshade relief map of Mullins Glacier from airborne LiDAR (2 m resolution) showing ASD. Chronological tie points for ASD 1 and ASD 6 are derived from cosmogenic-exposure dating of surface clasts as reported in ref. 14. (**b**) Additional age control for Mullins Glacier inferred from integrating the modern surface flow velocity^13^ (smoothed with a 400 m running average) (black line) as a function of distance down valley. The grey dashed lines represent the conservative bounds on this age estimate, computed using the mean velocity and the instantaneous surface velocity^14^. Inferred ages correspond well with tie points derived from cosmogenic-exposure age studies; red horizontal lines show modelled ages and age scatter in cosmogenic data at those sites^14^. (**c**) The modelled output for the onset and development (thickening) of supraglacial debris over the past 250 ka in the critical zone (at L02) that ultimately produces inclined debris layers (IDLs)/ASDs; curved bars reflect the non-linear reduction in the rate of debris development over time, which arises because thicker debris slows underlying ice sublimation; the width of the bars refers to the duration of modelled negative mass balance; modelled thicknesses match well with observed thicknesses seen in hand-dug exposures and ground-penetrating radar. The numbers correspond to the ASD/IDL number. (**d**) Modelled point mass balance in the critical zone (L02) over the past 250 ka; red shading highlights negative mass balance corresponding with modelled development of supraglacial debris in the critical zone. (**e**) Integrated summer energy^31^,^32^ using a threshold of 250W  m^−2^. (**f**) Temperature departure from modern conditions as recorded in the Taylor Dome core^4^. Marine isotope stages are labelled for reference. (**g**) Annual accumulation rate at Taylor Dome^4^; data in **f**,**g** are used to produce paleo precipitation and temperature data for Mullins and Friedman glaciers (see ‘Methods' section). (**h**) For reference, temperature departure from modern conditions recorded in the Vostok ice core^3^. The results show that modelled ASD/IDL growth is correlated with peaks in integrated summer energy; this occurs when solar insolation forcing amplifies the weaker obliquity-paced atmospheric temperature forcing.

We next reconstruct broad changes in mass balance—and associated IDL formation periods—in the critical zones of Mullins and Friedman glaciers during late Pleistocene time. To accomplish this, we repeat our modelling exercise but this time use proxy data (inferred precipitation and temperature) from nearby Taylor Dome (<40 km distant)^4^ and reconstructed high-latitude insolation forcing from Berger and Loutre^30^. These data are combined with our measured micrometeorological data (Supplementary Fig. 1) to produce synthetic meteorological input representative of specific paleo-environmental climate conditions (see ‘Methods' section). The modelling results show a distinct ∼41 ka periodicity for mass balance change in glacier critical zones (Fig. 4 and Supplementary Fig. 4), even though proxy records for precipitation and temperature at Taylor Dome show a relatively weak response to this ∼41-ka forcing. This implies that mass-balance loss and IDL formation over this time period includes complex feedback mechanisms and is paced most strongly by total summer energy (Fig. 4), defined as the sum of insolation intensity exceeding a given threshold^31^,^32^, rather than peak summer insolation, atmospheric temperature or precipitation changes alone (Fig. 3).

A chronology for Mullins Glacier, based on cosmogenic-nuclide exposure ages of surface clasts and the integration of modern, horizontal ice-surface flow velocities for both Mullins and Friedman glaciers^13^,^14^ (see ‘Methods' section) is in accord with obliquity-paced mass balance changes (Fig. 4). These data, when combined with the morphological consistencies among both glaciers, and the mass balance modelling results, show that the observed IDLs and corresponding ASDs are Pleistocene in age, ranging from ∼12 ka BP for IDL 1 and ASD 1 near valley heads to ∼210–230 ka BP for IDL 6 and ASD 6 several kilometres down valley (Figs 1 and 4). As a partial test of the obliquity-paced IDL formation model, we coupled resolved changes in mass balance with a model for the development of supraglacial debris in the critical zone. Results from this modelling exercise show that each major peak in negative mass balance is capable of producing the requisite thickness of debris layers in the critical zone that is consistent with estimated IDL thickness from GPR and ice core data (Fig. 4; see ‘Methods' section).

## Discussion

These results highlight the sensitivity of cold-based debris-covered alpine glaciers to obliquity forcing in a region where ∼100 ka forcing dominates most other Pleistocene climate archives^2^,^3^,^4^,^5^,^6^. Although several environmental factors influence mass balance history (Fig. 4d), we demonstrate that for Mullins and Friedman glaciers the forcing from obliquity-paced changes in solar insolation, perhaps exacerbated by high atmospheric transmissivity of the region^33^, is sufficient to cause non-linear geomorphic responses that result in the formation of repetitive IDL and ASD. The influence of air temperature, precipitation and solar insolation are all manifest in the modelled mass balance history (Fig. 4d), but these environmental factors amplify/weaken in complex ways: insolation serves to amplify the effect of temperature forcing so that when combined with changes in precipitation, it imparts a non-linear response in ablation that is paced at the obliquity timescale.

More broadly, these results highlight the importance of critical zone ice dynamics in the development of cold-based, debris-covered glaciers. Similar-appearing ASDs on other debris-covered glaciers in Antarctica^34^,^35^,^36^, and even at lower latitudes^19^,^25^, may also record cyclical changes in climate. Although it is clear that not all changes in the surface morphology of debris-covered glaciers (nor changes in englacial debris content) are related to climate variation, for example, refs 37, 38, our model framework provides a new and testable hypothesis for further exploration.

Finally, our conclusion that highlights the importance of integrated summer energy in high-latitude glacial dynamics may also have implications for the dynamics of nearby blue ice areas in Antarctica^39^, especially given their high ablation sensitivity to solar insolation^40^, and possibly even for postulated debris-covered glaciers on Mars. For the latter, imagery from the High Resolution Imaging Science Experiment^41^ and Context Camera^42^ show spoon-shaped hollows at the head of mapped debris-covered glaciers on crater-interior walls^22^,^43^; many such features also show arcuate surface ridges in ice-ablation areas reminiscent of ASDs^44^,^45^,^46^. If correct, then it suggests the tantalizing notion that climate signals may be recorded in the surface topography and englacial structure of cold-based, debris-covered glaciers on Mars^47^,^48^,^49^, and that the distribution of relatively clean glacier ice and debris-rich ice in these systems may likewise be resolved^22^.

## Methods

### Modelling overview and procedure

We investigate mass balance within the critical zone of Mullins and Friedman glaciers (Fig. 1) with an EBM driven with (1) modern conditions, (2) single parameter (atmospheric temperature, snowfall, solar insolation) perturbations from these modern conditions and (3) conditions that are representative of local climate conditions over the past ∼230 ka. The EBM is then coupled to a model for the development of supraglacial debris layers (and subsequent IDL) in the critical zone. We do not include variations in ice flow or the net/spatially distributed mass balance over the entire glacier.

To accomplish this, high-temporal resolution (minimum ∼1 h) meteorological data are required to model small (cm scale) changes in point mass balance. This is particularly true for glacial systems that are strongly impacted by diurnal meteorological forcing^50^. For Mullins and Friedman glaciers, modern meteorological data can be measured at a variety of prescribed resolutions, but these data must be simulated for past climate regimes. As described below, we accomplish this through directly altering, with a set of transfer functions based on paleoclimate data, several years of measured modern meteorological data to represent past conditions. With this simulated meteorological data set, we then execute the EBM (at a one hour time step over our simulation period of 12 years) to compute the estimated average annual point mass balance for any time (and associated climate configuration, for example, orbital forcing) in the climate record.

We compute the annual average mass balance at two locations within the critical zone of Mullins Glacier. Location one (L01) is located immediately adjacent to the primary meteorological station (AWS01), and is ∼100 m down valley from, and 15 m lower, than the modern equilibrium line. L01 is used to validate our model under modern conditions; it is also used to test the relative magnitude of accumulation/ablation forcing and its resulting impact on mass balance in the critical zone for past climate configurations. When modelled over conditions representative of the past 230 ka, the mass balance at L01 shows cyclical fluctuations in magnitude, but remains negative at each time step. We modelled a second location (L02), situated ∼400 m up-valley and ∼100 m higher than L01 (for example, within the modern ice-accumulation area) and adjust L02's meteorological parameters from those measured at L01 by including (1) a constant air temperature decrease of 1 °C according to an average lapse rate of 9.8 °C km^−1^ (ref. 51), and (2) an increase in annual snowfall by 6 cm (to reflect the observed steep gradient in wind-blown snow accumulation that increases toward the headwall).

Starting at 230 ka BP and moving forward in time at 0.5 ka time steps, we couple the mass balance results for L02 to a geomorphic model for the development of supraglacial debris/IDL under conditions of negative mass balance (see below). The geomorphic model computes the accumulated thickness of supraglacial debris over time. When the mass balance at L02 returns to a positive value, we assume burial of the supraglacial debris layer (following a small time lag to account for enhanced ablation of new snowfall due to the lower albedo of the rocky debris surface, see below).

The following sections provide details on the above model framework, and include additional data for site conditions, meteorological records, meteorological transfer functions, numerical models used and model validation.

### Modern micrometeorological data

Meteorological conditions across Mullins and Friedman glaciers were monitored from 2008–2013 using automatic weather stations (AWS) at four locations^12^ (Fig. 1). These stations recorded (1) total downwelling (global) short-wave solar radiation; (2) surface barometric pressure; (3) air temperature and relative humidity (RH) at ground level (5 cm) and mast height (1.8 m); and (4) mast height wind speed and direction. Each station sampled environmental conditions every 3 min and logged averages at 15 or 30 min intervals, for example, ref. 52. In addition, for the period 2010–2013, AWS01 (77.9096°S, 160.59789°E; 1,642 m MSL), located within the critical zone on Mullins glacier, employed a Kipp and Zonen CNR 4 net radiometer installed at mast height to record (6) total downwelling (global) short-wave solar radiation (enclosed thermopile pyranometer; measurement range of 0–2,000 W m^−2^ and a resolution of <1 W m^−2^), (7) upwelling short-wave solar radiation (enclosed thermopile pyranometer; measurement range of 0–2,000 W m^−2^ and a resolution of <1 W m^−2^), (8) downwelling longwave radiation (enclosed thermopile pyrgeometer, measurement range of −220 to +200 W m^−2^), (9) upwelling longwave radiation (enclosed thermopile pyrgeometer, measurement range of −220 to +200 W m^−2^), and (10) internal CNR4 housing temperature (Pt-100 thermistor). For this study, we use data from AWS01 during the period from 01/01/2011 to 12/31/2012 (Supplementary Fig. 1). This two-year time interval displays the greatest overlap with nearby meteorological stations and shows minimal gaps due to intermittent sensor failure. On the basis of the strong consistency among data recorded at AWS01 and nearby AWS03, which itself has a 9-year continuous data set, as well as the minimal variation noted in annual weather conditions monitored at nearby long-term weather stations (∼30 years continuous data)^51^, we conclude that our 2-year data inventory from AWS01 well represents modern conditions at our site. In addition, the strong similarity in data from AWS01—located in the critical zone of Mullins glacier, and AWS04 located at the same elevation in the critical zone of Friedman glacier (Fig. 1)—indicates that AWS 01 is representative of the critical zone meteorology on both glaciers.

To assess aerodynamic roughness near AWS01, we installed a vertical array of four Onset Computer Corporation cup-style anemometers spaced at roughly logarithmic heights of 0.28, 0.66, 1.34 and 2.2 m, ∼3 m from AWS01; this array sampled data every 30 s, stored at 5  min intervals, for average wind velocity, maximum gust velocity and wind direction (top anemometer only) for the period 11/29/2010 to 12/09/2010. Wind data were processed with a 15-min running average to reduce noise.

### Modern snowfall

Snowfall in Mullins and Friedman valleys is sourced from a combination of (1) direct atmospheric precipitation, typically associated with coastal storm tracks, and (2) wind-transported snow sourced from the East Antarctic Ice Sheet (EAIS)^15^. In December 2010, we deployed a series of time-lapse cameras to capture the frequency and approximate magnitude of snowfall events in and around the critical zone of Mullins Glacier. The time step of repeat images ranged from 1 to 6 h, depending on the season. Due to intermittent instrument failure and the lack of light in the polar winter, the data are discontinuous across the 2-year deployment period (01/2011–01/2013). Supplementary Fig. 1 shows our snowfall record for the measurement period. To convert fresh snowfall to cm water equivalent, we used the average of several density measurements (104 kg m^−3^) of fresh snowfall collected on site during the 2011 and 2012 field seasons.

### Modern ice ablation

We measured ablation directly by installing an array of 1.25-inch PVC ablation stakes in the uppermost ablation areas of both Mullins (11 stakes, installed 2010) and Friedman glaciers (5 stakes, installed 2011)^12^. Stakes were measured annually in mid-December for repeat analysis of (1) ice loss (the measured distance from top of the stake to ice surface) and (2) geographic position. All stakes were installed on bare ice. Geographic positions for each stake were computed with a Trimble 5700 global positioning system, a Zephyr geodetic antenna and utilized a minimum 50-min residence time. Post processed kinematic differential location correction was performed with data from a base station located ∼7 km away on University Peak (77.86259°S, 160.75983°W; 2,195.3 m). Results over a 3–4-year baseline indicate that the modern ablation of exposed glacier ice in the uppermost ablation areas on Mullins and Friedman glaciers average 5.56 cm a^−1^ (*σ*=1.86) and 6.86 cm a^−1^ (*σ*=2.1), respectively, with the stake located closest to AWS01 averaging a slightly higher ablation rate of 7.1 cm a^−1^. Horizontal displacement down valley for all stakes averaged 9.2 cm a^−1^ for Mullins and 10.1 cm a ^−1^ for Friedman glaciers.

### Surface roughness

We used our vertical wind-profile measurements to derive an estimate for the local mean aerodynamic roughness (*z*_0a_) at AWS01 (Supplementary Fig. 5). Data points were iteratively fit to a logarithmic profile to determine *z*_0a_, computed as the ordinal intercept of the best fitting line (in a least squares sense) of ln(*z*-*d*) verses *u*(*z*) where *z* is the measurement height, *d* is the displacement height (∼0 m), and *u*(*z*) is the wind speed at height *z*. This resulted in a computed mean aerodynamic roughness of *z*_0a_=0.0065, m over the sampling period. This value is higher than values for *z*_0a_ typically reported for ablating glacier ice and snow areas (see review in ref. 53, Table 2), but there are two likely reasons for this. First, surface debris (length scales of ∼0.1–1 m) is present in the general vicinity of AWS01 on Mullins glacier. Second, due to a lack of co-located temperature profile measurements with our wind-profile measurements, we are unable to apply corrections for atmospheric stability in our *z*_*0a*_ estimate and have thus implicitly assumed neutral stability during the measurement period. This assumption requires that our mean *z*_*0a*_ estimate should be considered a maximum estimate due to the likelihood that conditions were skewed toward atmospheric boundary layer stability (the typical condition for glaciated regions in the summer) during our measurement period.

In calculating *z*_0a_, we made the following omissions. Only data for which the wind speed at the lowest anemometer was >2.5 m s^−1^ are included. This segregation was done to minimize the potential errors associated with the thermal instability of the atmosphere, and to use only data for wind speeds at which mechanical turbulence exceeded buoyancy effects^54^,^55^. Measurements were also discarded if: (1) zero wind speeds were recorded by any anemometer, (2) inverted wind speeds were recorded (higher anemometer recording a lower velocity than a lower anemometer) or (3) wind speeds at the highest anemometer were <3 m s^−1^.

### Surface albedo

Albedo (*α*) is not constant, but rather depends on surface cover (fresh snow, firn, bare ice, supraglacial debris and so on.) and zenith angle^56^,^57^. To determine the albedo of bare ice at the location of AWS01, we take the ratio of measured upwelling shortwave (*K* ↑) to global downwelling shortwave (*K* ↓) radiation 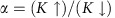 at all time steps in the selected 2-year record. We (1) eliminate those measurements taken at the lowest solar azimuth angles (all winter measurements and all records that are >5 h from solar noon), (2) disregard records within 10 days following a fresh snowfall event and (3) compute the mean (0.45). We purposely bias our albedo estimate toward values near solar noon (highest zenith angles) because the sensitivity of the land surface energy balance to albedo is the highest during periods of high *K*↓. We disregard the data following fresh snowfall events in order to include in the mean of only those measurements made over bare ice. We do not model the zenith angle dependency of albedo. We address the modelled albedo change through time as a function of changing surface type below.

We set the albedos of fresh snowfall and firn to the mean of published values (fresh snow *α*=0.87, firn *α*=0.53) as reported by ref. 58. We do not explicitly model the energy balance of debris-covered ice and therefore do not require an estimate of its albedo.

### Paleo proxy data

To derive representative paleoatmospheric temperatures and past ice-accumulation rates for Mullins and Friedman glaciers, we use the δ^18^O, δD and ^10^Be records from the ice core recovered at nearby Taylor Dome (TD) (45 km distant), referenced to the st9810 timescale^4^,^59^. The δ^18^O record is converted to temperature deviation from modern conditions according to the calibrations of Steig, Morse^4^ and Waddington and Clow^60^. Past accumulation rates are determined from the concentration of ^10^Be, using relationships between the measured concentration and flux^4^, with flux estimates of Steig, Stuiver^61^. The top of atmosphere (TOA) paleo solar insolation values at 77°S were determined according to the method of Huybers^32^ using the orbital configuration parameters of Berger and Loutre^30^.

### Transfer functions to derive paleo micrometeorological conditions

In order to execute the EBM under estimated paleoclimate conditions, we require a set of transfer functions for each input parameter (air temperature at mast height, precipitation, longwave downwelling radiation, total shortwave downwelling radiation) at our input time step (1 h) from the available proxy data. In the following sections, we develop equations of the form 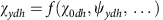 where *χ* is the parameter of interest and *Ψ* is a paleo proxy. The subscripts index time such that *χ*_*ydh*_ represents the value of *χ* at year *y* before present (BP), day of year *d*, and hour *h*. The notation *χ*_0*dh*_ represents the modern (at year zero BP) measured values for the parameter of interest.

### Paleo air temperature

We estimate past mast height temperatures (*θ*_*ydh*_) by assuming a simple linear relationship between the variation in temperature at AWS01 on Mullins Glacier and the average annual atmospheric temperature deviation at Taylor Dome. We apply a constant adjustment to each measurement point such that


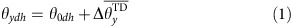


where *θ*_0*dh*_ are our measured data and 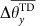 is departure, at year *y*, in annual average temperature at TD from modern conditions.

### Paleo snowfall

To estimate past snowfall (*P*) in a format suitable to our EBM approach (*P*_*ydh*_), we assume that the total annual precipitation (including atmospheric precipitation and wind-blown snow) at the headwall of Mullins and Friedman glaciers is linearly related to the average annual net ice accumulation rate at TD (in water equivalent). Implicit in this approximation are the assumptions that (1) net accumulation at TD is directly proportional to precipitation at TD (that is, there is negligible variation in ablation at TD), and (2) that fluctuations in precipitation at TD co-vary with precipitation (including wind-blown snow) at our nearby study site. We preserve the timing and duration of our measured snowfall events and alter only their magnitude (that is, total precipitation per precipitation event) such that the precipitation is adjusted with the function.


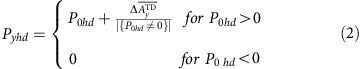


where *P*_*0dh*_ are the measured precipitation data, 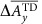
is the departure, at year *y*, in annual TD accumulation from modern conditions. The denominator in the fraction represents the total number (cardinality) of measurement hours where non-zero precipitation occurs.

### Paleo downwelling longwave radiation

Downwelling longwave radiation (*L*↓) is a function of the integrated temperature of the atmospheric column and its effective emissivity. We estimate paleo values of *L*↓_ydh_ for our model by directly adjusting our modern measured *L*↓_0dh_ based on changes in temperature, *θ*_*ydh*_. To do this, we adopt the Brutsaert^62^ parameterization using coefficients optimized to our location (derived from computing a best fit to our measured data through the minimization of the summed square of residuals adjusting the weight of outliers with bisquare weights), determine the difference in the predicted *L*↓ between modern conditions and a prescribed paleo atmospheric condition, and then adjust our measured *L*↓_0*dh*_ by this amount to arrive at *L*↓_*ydh*_. This procedure is represented with the equations


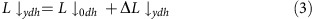


And


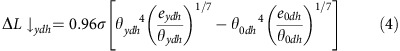


where *σ* is the Stefan-Boltzmann constant (5.67 × 10^−8^), and *e* is the actual vapour pressure at screen height, and *θ*_*ydh*_ is determined from equation (1). The values for vapour pressure were determined from screen height temperature (*θ*) and the dewpoint using the approximation of ref. 63, which is most appropriate for cold temperatures^64^. The dewpoint is determined from the measured relative humidity (RH) using a Magnus formula. Implicit in this overall approach to estimating *L*↓_*ydh*_ is the assumption that RH under previous climate conditions was similar modern values.

### Paleo downwelling total shortwave radiation

To estimate past intensity and seasonality of downwelling shortwave radiation (*K*↓) at ground level, we first compute the difference between the modern and past daily average top of atmosphere (TOA) solar insolation, denoted 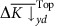, as


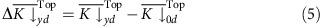


where 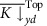
is the daily average TOA insolation at year *y* BP on day *d*, and 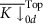
is modern TOA insolation on day *d*. We then modulate our modern measured hourly insolation values (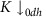) by the change in the daily average TOA insolation multiplied by a reduction factor that represents the estimated daily atmospheric transmissivity (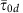) to arrive at


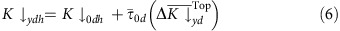


where 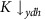 is estimated hourly shortwave insolation values at year *y* BP for input into our EBM framework. To determine 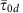, we first compute the average clear-sky transmissivity for the study region under modern conditions. Using two years of measured *K*↓ from station AWS03 located 3 km down valley (to avoid shading from valley sidewalls) (Fig. 1), we compute daily average insolation, isolate clear-sky days from the record, and take the average of the ratios of ground-based to TOA daily insolation values (Supplementary Fig. 6). Using this value as our annual average clear-sky transmissivity, we use the function of ref. 65 to modulate this annual average:


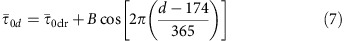


where 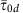 is the estimated modern daily atmospheric transmissivity factor, 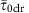 is the measured average annual atmospheric transmissivity (0.71) fit to 2 years of measured data, and *B* (0.02) is an amplitude factor^65^ that best fits our data.

### Surface energy balance model overview

We implemented a surface energy balance model (EBM) using source code derived from the open-source DEBAM of Hock and Holmgren^29^ and Reijmer and Hock^66^ designed specifically for glaciated environments. Below, we give a brief overview of the EBM approach within DEBAM and highlight DEBAM customizations and routines specific to our implementation. We did not execute the EBM in a spatially distributed manner, but instead utilized its routines to investigate point mass balances.

Within the EBM, all components of the radiation balance and heat fluxes are estimated at each time step to address the governing energy balance equation:


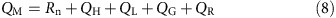


where *Q*_M_ is the energy available for melt, *R*_n_ is the net radiation, *Q*_H_ is the sensible heat flux, *Q*_L_ is the latent heat flux, *Q*_G_ is the ground heat flux, and *Q*_R_ is the sensible heat flux supplied by rain. We disregard *Q*_R_ due to climate conditions that prohibit rainfall at our study location^15^. The remaining fluxes are calculated using measured or simulated (see section 3) meteorological data at each time step. We assume *Q*_M_ ≈ 0 for our system, but do not enforce this constraint.

The net radiation is defined as 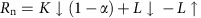 where *K*↓ is the downwelling shortwave solar radiation, *L*↓ is the downwelling longwave radiation, and *L*↑ is the upwelling longwave radiation from the snow/ice surface. *K*↓ and *L*↓ are from prescribed meteorological inputs. *L*↑ is equal to 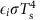, where *T*_s_ is the modelled surface skin layer temperature determined from the ice/snow temperature and density model^66^,^67^ (see below). The albedo term is dynamic and responds smoothly to surface state transitions according to the model of ref. 68. This albedo model estimates (1) how quickly the albedo transitions from that of fresh snow to that of firn after a snowfall event and (2) how the albedo changes to that of bare ice when the snow depth becomes thin.

The turbulent heat fluxes *Q*_H_ and *Q*_L_ are computed following a bulk aerodynamic approach^69^ incorporating atmospheric stability correction factors based on Monin-Obukov theory. The stability functions for momentum, moisture and heat are calculated according to the Businger–Dyer expressions, for example, ref. 70, for the unstable case, and according to Beljaars and Holtslag^71^ for the stable case—with the stability length scale computed by iteration^29^ at each time step. Under highly stable atmospheric conditions and/or wind speeds below the minimum anemometer threshold, the stability length scale was set to 0.3 to maintain model stability. Both downward and upward energy and mass fluctuations (sublimation/resublimation) are tracked for each time step. The required roughness lengths for temperature (*z*_0θ_) and vapour pressure (*z*_0e_) are computed from the measured value for aerodynamic roughness length (*z*_0a_) based on the procedure of ref. 72 and ref. 73. Vapour pressure at the snow/ice surface is assumed to be equal to the saturation vapour pressure.

The surface temperature (*T*_s_) and ground flux components are calculated from the multi-layer ice/snow temperature and density model of ref. 67. The model solves, on a vertical grid extending from the surface, the thermodynamic energy equation


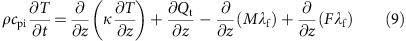


where *c*_pi_ is the heat capacity of ice, *T* is the temperature, *κ* is the effective conductivity, *Q*_t_ is the combined contribution from the turbulent and radiative fluxes at the surface, *M* is the melt rate, *λ*_f_ is the latent heat of fusion, and *F* is the refreezing rate. The effective conductivity (*κ*) characterizes the energy exchanges through convection, conduction, radiation and vapour diffusion, and is computed according to ref. 74 as a function of density. The density of any snow layer, if present, is determined by the snow densification model of ref. 75. Following the calculation of an initial temperature profile, the melt/refreezing amounts—if any—within each snow layer are calculated according to the temperature and water content of the layer. If water is present in the system (from surface or internal melt) it is allowed to percolate downward through snow layers at a rate that is controlled by an irreducible water content computed according to ref. 76 as a function of snow density. The surface skin temperature (*T*_s_) is determined from a linear interpolation of temperatures of the uppermost two layers. To maintain stability within the overall EBM, the multi-layer ice/snow temperature and density model is executed at a time step of 1/100 of the main execution time step as determined by the temporal resolution of the meteorological input data (1 h). Vertical layers are added to the model as snow accumulates via precipitation and are removed as a function of modelled ablation.

### EBM mass balance change

The model includes both positive (resublimation) and negative (melt and sublimation) mass fluxes to compute the total ablation. At each time step, these are determined from the surface temperature (*T*_s_), the latent heat term (*Q*_L_) and the energy available for melt (if any) (*Q*_M_), represented by the residual in equation (8). If *T*_S_ is <0 °C, then ablation (*A*) (in units of mm water equivalent time step^−1^) is entirely accommodated through sublimation/resublimation as 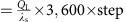, where *λ*_S_ is the latent heat of sublimation in units of J kg^−1^, step is the model time step and the direction of latent heat flux (the sign of *Q*_L_) determines if mass is lost or gained in the system. If 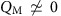, then the use of the extra energy in ablation (*A*) is determined by


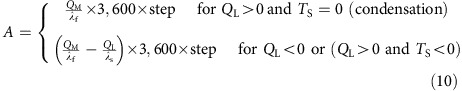


where *λ*_f_ is the latent heat of fusion in units of J kg^−1^. The point mass balance (MB) is then simply the accumulation combined with the ablation in units of water equivalent.

### EBM initialization

The surface condition at the start of each model run is assigned as bare glacier ice. For model validation under current conditions (see below), we initialize the ice-surface temperature (*θ*_s_) as 262°K from 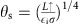 where *L*↑ is the measured longwave upwelling at time *t*=0, *σ* is the Stafan–Boltzman constant and *ɛ*_i_ is the emissivity of ice (0.99). For model runs under synthetic paleo conditions, we set the ice-surface temperature to be equal to the air temperature at hour zero (*θ*_*y*00_). We initialize the subsurface temperature profile using model results of Mackay *et al*.^12^, (based on measured borehole data collected in 12/2009). When simplified to fit a piecewise-linear model required by the EBM, the initial temperature depth profile takes the form of three linear segments defined by the temperature points 262°K, 266°K, 249°K and 273°K at corresponding depth points 0, 1.2, 15 and 180 m, respectively.

Before the model is used to estimate MB, it is executed for an 8-year spin-up period in order to better tune subsurface temperatures to the model scenario. During the spin-up period, the model is driven with the meteorological parameters (concatenation of the 2-year data set) that will be used in the model scenario.

### EBM validation

When executed using modern meteorological conditions and tuned to measured initial conditions (Supplementary Fig. 1), the EBM model results (Supplementary Figs 7,8) broadly agree with other energy balance studies in the region, for example, refs 40, 77, as to the magnitude, direction and seasonal timing of major energy flux components. To validate the effectiveness of the EBM in capturing the physical dynamics of our system, we directly compare modelled surface temperatures and ablation rates against measured surface temperatures and ablation stake data at L01 (Supplementary Figs 9,10). Surface temperatures agree within a mean absolute error of 1.4 °C over the full time period. Importantly, the error drops to MAE=∼0.5 °C during the summer period when the majority of ablation occurs. Modelled mean annual point mass balance at L01 (6.7 cm a^−1^) agrees well with the collocated ablation stake at L01 (7.1 cm a^−1^) and within margin of the measured mean of all ablation stakes in the critical zone of Mullins Glacier (5.56 cm a^−1^). Finally, when large (>2 cm snow depth) snowfall events occurred over bare ice, we use the time-lapse camera record to confirm the modelled dynamics of snowpack evolution and ablation. Although limited in temporal resolution, this exercise demonstrates excellent agreement between the observed and modelled ephemeral snow pack ablation rate.

### Debris layer thickness model

Following Mackay and Marchant^14^, we model the development of a layer of supraglacial debris using the framework of a 1-D column of ablating dirty ice. Within this model domain, the supraglacial debris layer will (1) increase in thickness from the base at a rate that is proportional to the sublimation rate and the concentration of englacial debris (with debris thickness increasing at a faster rate with higher concentrations of englacial debris and higher ablation rates), and (2) decrease in thickness at the surface via steady erosion. Further, if located within the rockfall runout zone^12^, the supraglacial debris layer will gain additional thickness from direct rockfall accumulation. This is represented as


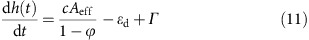


where *h(t)* is the thickness of the supraglacial debris layer at time *t*, *c* is the englacial debris concentration (by volume), *A*_eff_ is the effective ice down-wasting rate, *ɛ*_d_ is the bulk erosion rate of the supraglacial debris, *ϕ* is the average bulk porosity of the resulting supraglacial debris and *Γ* is the average rockfall accumulation rate.

We approximate *ϕ* and *ɛ*_d_ as constants equal to 0.3 (ref. 10) and 20 cm Ma^−1^ (refs 14, 78, 79), respectively.

The rockfall accumulation rate (*Γ*) is spatially variable and depends on the distance from the headwall^12^. We estimate a minimum range for this parameter using 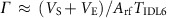, where *V*_S_ is the volume of supraglacial debris (3.4–3.9 × 10^5^  m^3^) estimate by integrating the measured supraglacial debris thickness^12^ across the valley lateral dimensions, *V*_E_ is the volume of englacial debris (2.8–8.4 × 10^5^  m^3^) estimate by multiplying the measured glacial ice volume^12^ by the average englacial debris concentration, *A*_rf_ is the estimated area of the rockfall accumulation zone (5.4 × 10^5^ m^2^) determined from GIS and the model results of Mackay *et al*.^12^, and *T*_IDL6_ is the estimated surface age at IDL 6 (215 ka)^14^. This calculation results in an average rockfall accumulation rate of ∼1–2 × 10^−3^  cm a^−1^.

The effective ice down-wasting rate (*A*_eff_) in equation (11) will change as a function of environmental forcing and the thickness of evolving supraglacial debris (*h*). As mass balance initially turns negative, incipient supraglacial debris (<∼1 cm) is likely sparse and would only impact ablation dynamics through localized changes in albedo. Under this scenario, we set the effective ice down-wasting rate equal to the modelled mass balance for the clean ice case (which includes the mass flux of snowfall). Once the debris layer evolves to become a continuous sheet, it is no longer appropriate to include mass gains from snowfall (because snow that falls on top of the debris tends to sublimate and not add to the glacier mass balance), and we equate the effective ablation rate to the modelled ablation (*A*) (MB minus snowfall). For continuous supraglacial debris thicker than a few cm, the ablation rate becomes vapour-diffusion-limited^80^ and the EBM model developed for clean ice is also not applicable. For such thick supraglacial debris conditions we abandon the EBM approach, for example, ref. 81, and instead follow Mackay and Marchant^14^ and model the ablation rate as an exponential function of the supraglacial debris thickness, for example, refs 82, 83, 84, 85, 86, using coefficients fit to experimental data from Mullins Glacier^10^. Thus, we arrive at a modelling scheme for the downwasting of ice that adapts to different debris thickness cases as follows:

1. For the case where ice is essentially bare, without debris cover, the downwasting rate is set equal to the EBM mass balance model results (which may include a positive ice growth contribution from precipitation).
2. For the special case where debris thickness=*H*_p1_≈1 cm, the downwasting rate is set to the bare-ice EBM mass balance results, except that accumulation from snowfall is ignored (that is, the EBM ablation rate, *A*).
3. Finally, for ice covered with debris thickness ≥*H*_p2_ ∼3 cm, the downwasting rate is determined by the (parameterized) vapour diffusion model.

This is represented as:


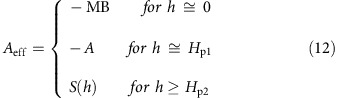


where MB is the EBM modelled clean-ice point mass balance (cm a^−1^), *h* is the debris thickness (cm), *A* is the EBM modelled ablation rate (cm a^−1^), *H*_p1_ is the first supraglacial debris thickness threshold (1 cm), *S*(*H*) is the ablation rate according to the exponential ablation model of Mackay and Marchant^14^ evaluated at debris thickness *h*, *H*_p2_ is the second supraglacial debris thickness threshold (3 cm).

The value of threshold #1 (*H*_p1_, 1 cm) is the approximate debris thickness at which a ‘continuous' debris cover is observed in the field. While fresh snowfall can persist and accumulate on bare ice/perennial snow banks, direct observations and time-lapse camera data (see ‘Methods' section) show that snowfall rapidly ablates off continuous debris covers. The value of threshold #2 (*H*_p2_, 3 cm) corresponds to the minimum approximate debris thickness at which the ablation of polar debris-covered ice can be reasonably represented by a vapour diffusion model. The exact value for this parameter is currently not well constrained. However, 3–7 cm is broadly indicated by the measurements/modelling of ref. 10; it is also a reasonable depth where direct sensible heat transfer is mostly eliminated. Choosing a value on the low end for this threshold range results in reduced modelled thickness of surface debris in critical zones. In doing so, we are imposing a conservative estimate to the thickness of the debris layers (and hence IDL) that can form. When modelled using a higher value for threshold #2 (for example, 7 cm), the modelled IDL thicknesses only increases by ∼20%.

In order to model the down-wasting rate for debris thicknesses between these defined debris thickness thresholds (i.e. for 0<*h*≤*H*_p1_ and *H*_p1_<*h*<*H*_p2_), we apply a simple linear ramp between each case in equation (12). When implemented in our numerical scheme this becomes:


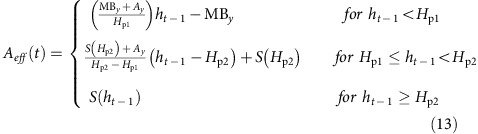


where *A*_eff_ is the effective ice down-wasting rate at the current time step, MB_*y*_ is the modelled clean-ice point mass balance at year *y* BP, *h*_*t-1*_ is the modelled debris thickness at the previous model time step, *S*(*H*_p2_) is the ablation rate according to the model of Mackay and Marchant^14^ evaluated for the debris thickness at threshold #2, *A*_*y*_ is the EBM ablation rate at year *y* BP, and *S*(*h*_*t-1*_) is the ablation rate according to the model of Mackay and Marchant^14^ evaluated for the debris thickness at the previous model time step. For the debris layer growth model, we utilize a numerical time step of 1 year.

Finally, we generate estimates for the thickness of supraglacial debris by simultaneously solving equations (11) and (13) in a forward modelling scheme.

### Chronological control

Chronological control is based on cosmogenic-nuclide exposure ages of surface clasts on Mullins Glacier, the integration of modern, horizontal ice-surface flow velocities for both Mullins and Friedman glaciers^13^,^14^ and the strong morphological consistencies between these two glaciers^12^.

As discussed in detail in Mackay and Marchant^14^, establishing tight chronological control derived from cosmogenic-nuclide exposure dates on surface clasts above buried glacier ice is non-trivial, with uncertainty arising from a host of pre- and post-depositional processes that may affect the nuclide inventory of individual samples. However, we place a high confidence on the cosmogenic-nuclide-derived age of (1) ASD 6 on Mullins Glacier (210–230 ka), which is based on the close spatial correlation of three separate samples, and (2) ASD 1 (∼12 ka), because the probability of erroneous ages decreases dramatically with decreased distance from the headwall^14^. We therefore use these dates as our high-confidence cosmogenic tie points. The dates and locations of additional samples^14^ fall between these two tie points and are consistent with our inferred chronology (see Supplementary Fig. 2).

As an independent approach to refine this chronology, we use measured horizontal surface flow velocities^13^, to derive age estimates of: Mullins ASD:ASD1: 10–13 ka, ASD2: 24–42 ka, ASD3: 41–72 ka, ASD4: 91–105 ka, ASD5: 130–175, ASD6: 196–290 ka. Friedman ASD:ASD1: 10–14 ka, ASD2: 22–55 ka, ASD3: 26–67 ka, ASD4: 37–80 ka, ASD5: 78–130, ASD6: 131–198 ka.

The large range in the above estimates is due to computing the surface age based on either (1) dividing the measured horizontal distance by the mean velocity of the entire region of interest or (2) integrating the measured surface ice-flow velocities at specific points along the glacier (smoothed with a moving average filter of 50 m) (see ref. 14). Both approaches are vulnerable to the assumption that modern flow velocity is a reasonable indicator of paleo-flow velocities (that is, that accumulation rate and environmental conditions have remained roughly constant) and that ice flow has been continuous over the time period of interest. Flow velocity was likely subtly different over the changing climate and mass balance regimes of the past ∼250 ka (due to ice hardness dependency on temperature; flow dependency on longitudinal stress of increased/decreased accumulation and so on.). Although the impact of past variations in flow velocity are difficult to predict, these age estimates are in accord with the high confidence cosmogenic-nuclide age tie points and are consistent with the proposed a ∼41 ka pulse cycle of IDL development.

### Data availability

The data that support the findings of this study are available from the corresponding author upon reasonable request. A subset of these data are available at http://people.bu.edu/marchant/databases/index.html.

## Additional information

**How to cite this article:** Mackay, S. L. & Marchant, D. R. Obliquity-paced climate change recorded in Antarctic debris-covered glaciers. *Nat. Commun.* **8,** 14194 doi: 10.1038/ncomms14194 (2017).

**Publisher's note:** Springer Nature remains neutral with regard to jurisdictional claims in published maps and institutional affiliations.

## Supplementary Material

Supplementary Information Supplementary Figures and Supplementary References.
