# Supplementary material for: Obliquity-paced climate change recorded in Antarctic debris-covered glaciers
Source: Nat Commun. 2017 Feb 10;8:14194. doi: 10.1038/ncomms14194 (PMC5309835; doi:10.1038/ncomms14194)
Supplement: Supplementary Information — Supplementary Figures and Supplementary References. [file ncomms14194-s1.pdf]

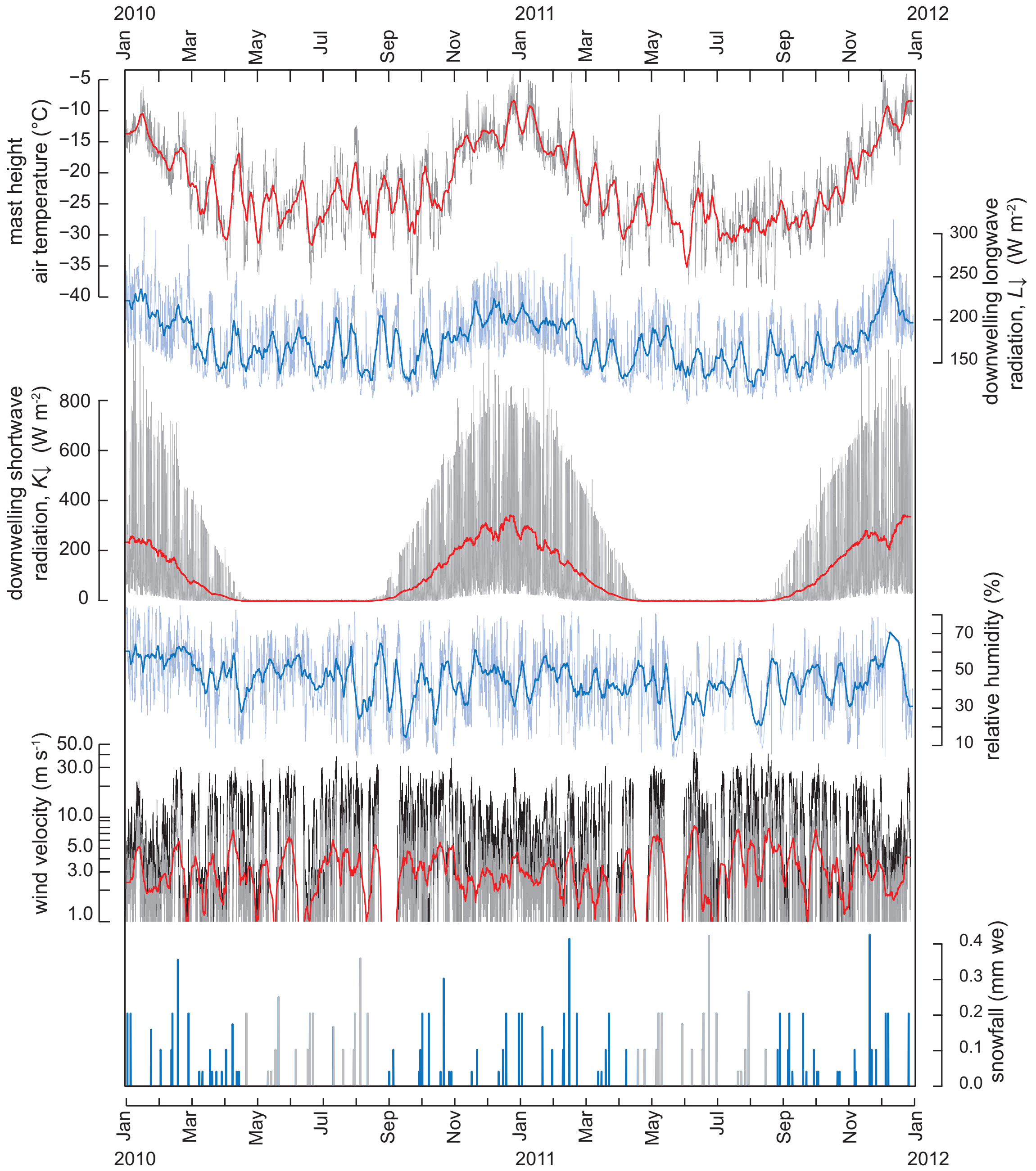

Supplementary Figure 1. Measured meteorological data from AWS01. For mast height air temperature (1.8 m) and downwelling shortwave radiation (top panels), thin grey lines show hourly data; the thick red lines plot 7-day running averages. For downwelling longwave radiation and relative humidity (mid-level panels), the light blue lines plot hourly data; the thick dark blue lines show 7-day running averages. Wind velocity (lower panel) is represented on a logarithmic scale; the thin black lines show gust velocity, thin grey lines show hourly mean velocity, and the thick red line is the 7-day running average. The snowfall record (bottom panel) is discontinuous across the measurement period due to lack of ambient light during the polar winter. Where gaps exist, we assume a similar frequency and magnitude of snowfall as for the measured periods; we fill the gaps with a synthetic record (grey lines) derived from the measured data (blue lines).

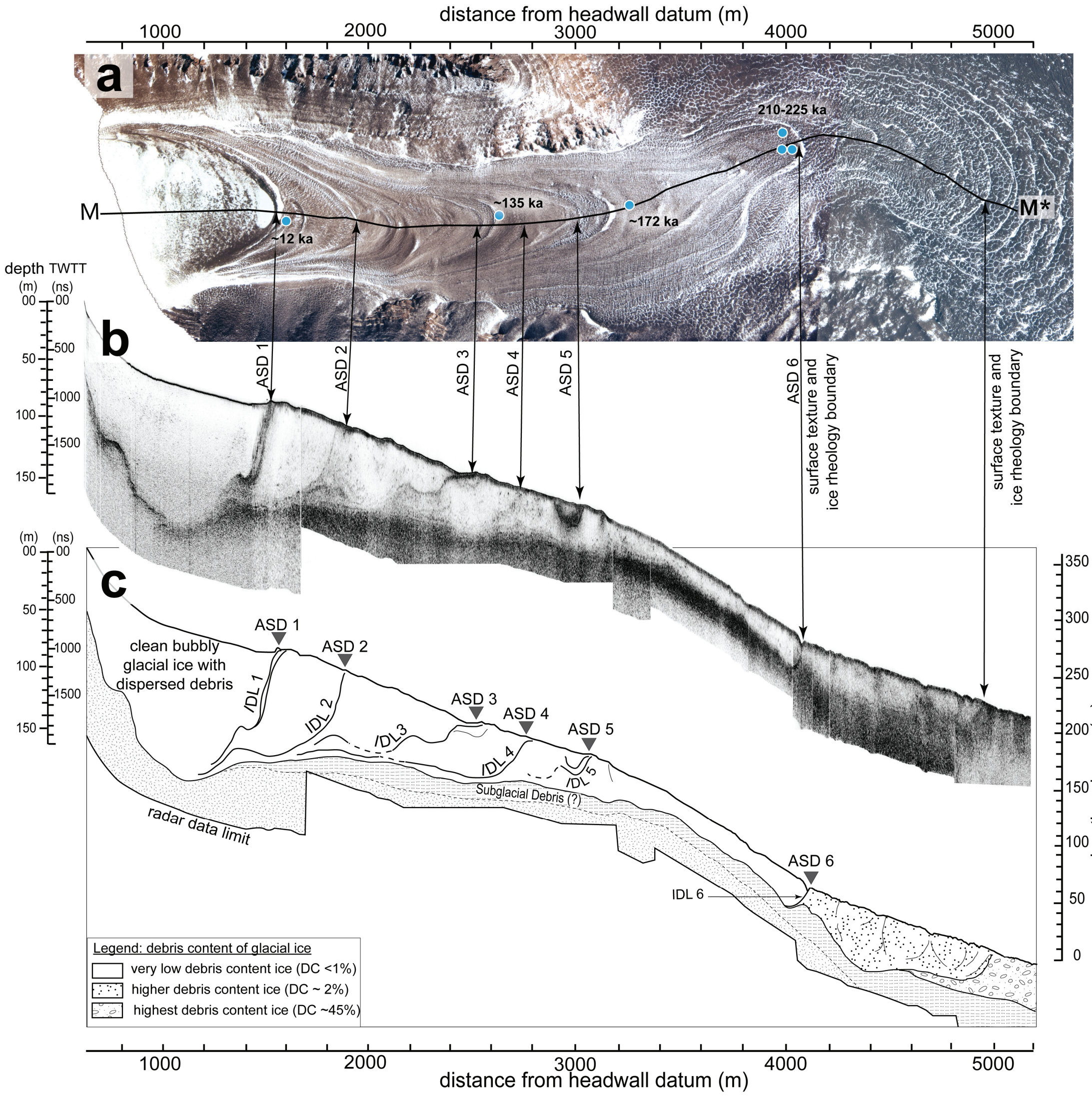

Supplementary Figure 2. Longitudinal GPR transect M – M* for Mullins Glacier. (a) vertical aerial photograph (USGS TMA 3080V0276) showing transect location. (b) radar data along line M-M*, collected with a Geophysical Survey Systems, Inc. Subsurface Interface Radar 3000 system, migrated, and post-processed with a Hilbert magnitude transform. (c) sketch of major radar reflectors and physical interpretation. The vertical depth scale for all panels is shown as two-way travel time (TWTT in ns) and as meters assuming an average radar travel time of 0.168 m ns^{-1}. Double arrowed lines between the top panel and the second panel mark the location of identified arcuate surface discontinuities (ASD) and highlight the spatial correlation between ASD and inclined debris layers (IDL). Data for the first ~900 m were collected using a 200 MHz antenna in distance mode; thereafter, data were collected with an 80 MHz antenna in point mode. Blue circles mark the location and age of cosmogenic nuclide samples [1] used to aid in the development of chronological control; modelled cosmogenic ages indicated next to each blue marker. Figure modified from Mackay, Marchant [2] Figure 6.

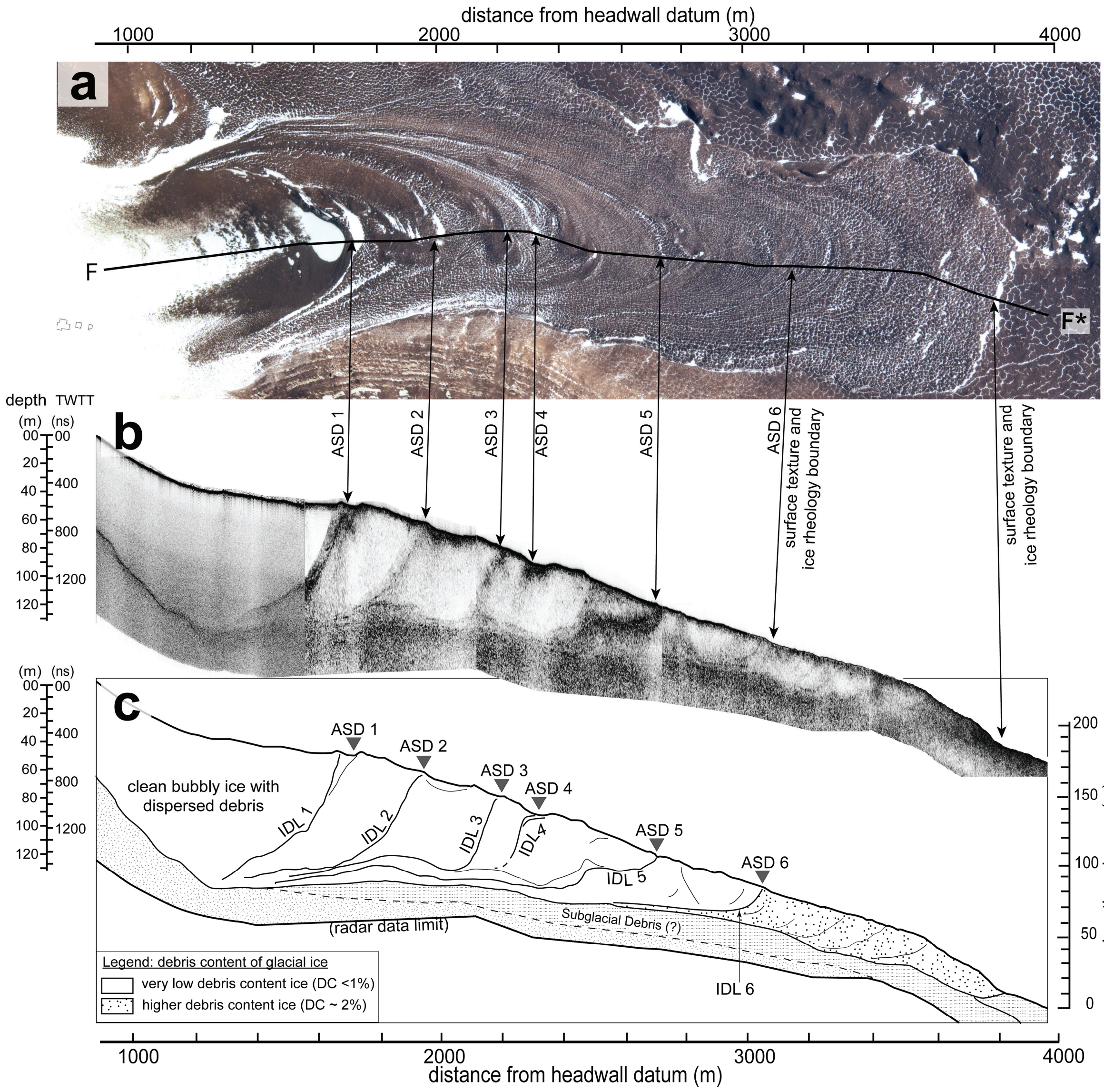

Supplementary Figure 3. Longitudinal GPR transect F – F* for Friedman Glacier. (a) vertical aerial photograph (USGS TMA 3080V0276) showing transect location. (b) radar data along line M-M*, collected with a Geophysical Survey Systems, Inc. Subsurface Interface Radar 3000 system, migrated, and post-processed with a Hilbert magnitude transform. (c) sketch of major radar reflectors and physical interpretation. The vertical depth scale for all panels is shown as two-way travel time (TWTT in ns) and as meters assuming an average radar travel time of 0.168 m ns-1. Double arrowed lines between the top panel and the second panel mark the location of identified arcuate surface discontinuities (ASD) and highlight the spatial correlation between ASD and inclined debris layers (IDL). Data for the first ~750 m were collected using a 200 MHz antenna in distance mode; thereafter, data were collected using an 80 MHz antenna in point mode. Figure modified from Mackay, Marchant [2] Figure 8.

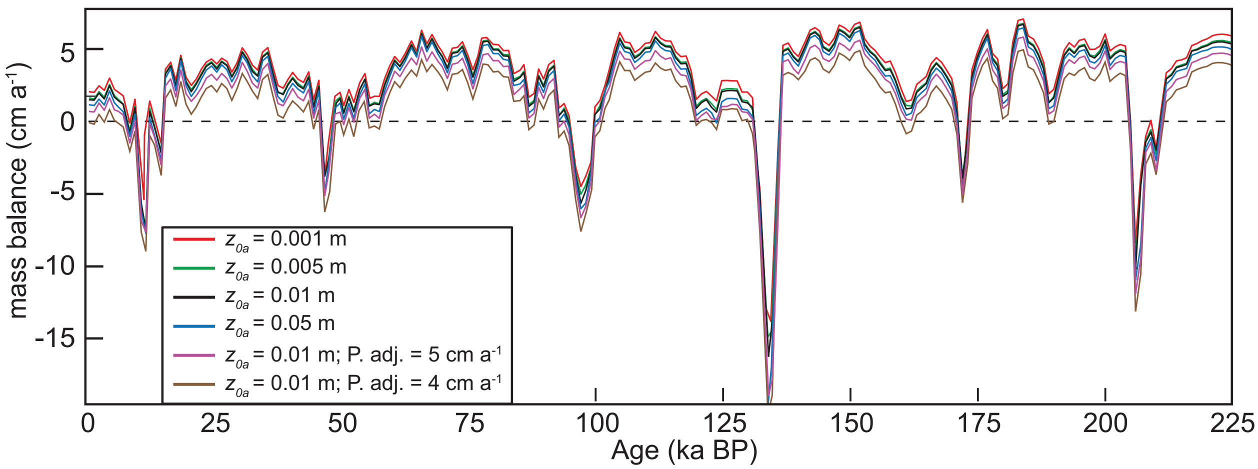

Supplementary Figure 4. Modeled point mass balance over the past 225 ka at location L02 evaluated over a range aerodynamic roughness (z_{0a}) values and snowfall adjustment factors (P.adj). Altering the value of z_{0a} changes the magnitude of the modelled mass balance (<~ 5%), but does not alter the timing or duration the major negative mass balance events (IDL causing events). The value z_{0a} = 0.065 m is used throughout this modelling study (see Methods). The effect of changing the snowfall adjustment factor (the value P.adj. = 6 cm a^{-1} is used through this study) used to model the precipitation at L02 (see Methods); perturbations to this parameter have little-to-no consequence for the main conclusions of this work

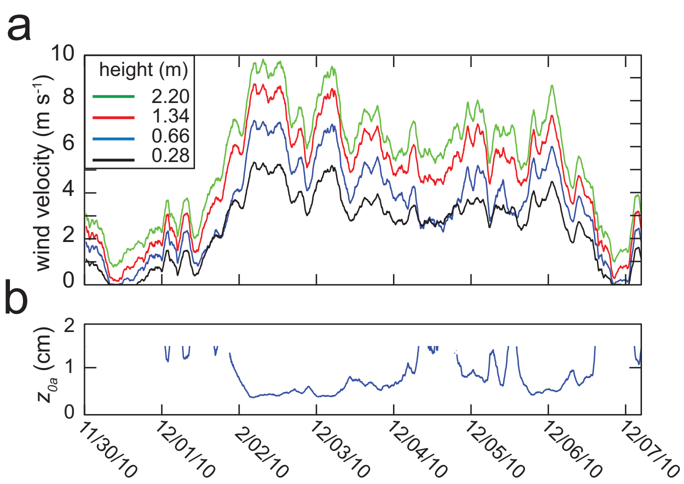

Supplementary Figure 5. (a) A portion of the measured vertical wind velocity profile at AWS01 (time scale shown in b). (b) Computed aerodynamic roughness (z_{0a}) for the wind profile of (a). Gaps that exist in the z_0 time series are due to periods of low wind or inverted wind profiles observed in (a) (see Methods. We compute a mean z_0 over the measurement period to be ~0.065m.

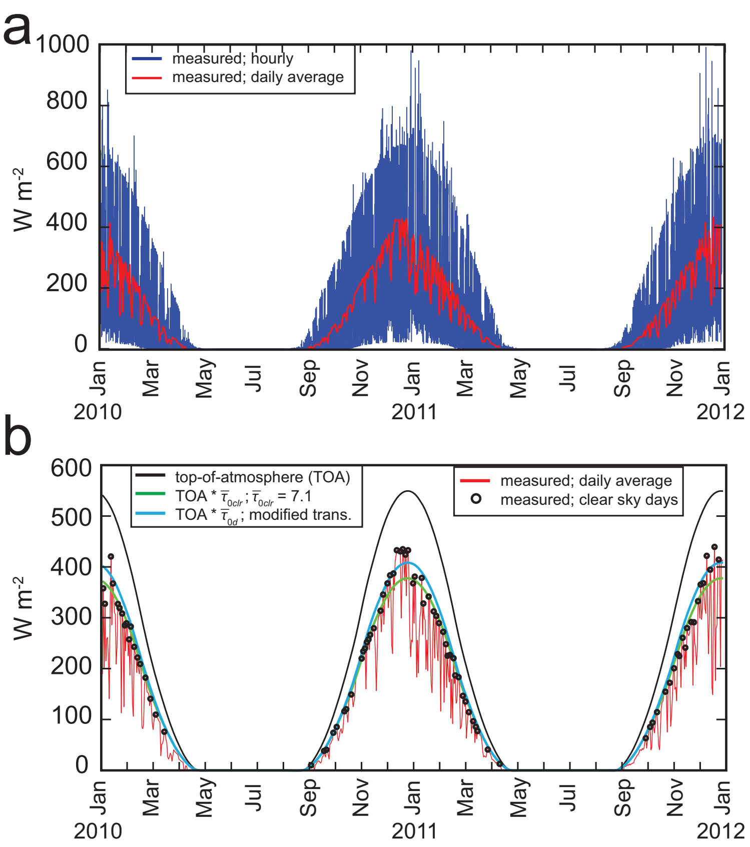

Supplementary Figure 6. Determination of shortwave atmospheric transmissivity. (a) Measured hourly (blue) and daily average (red) downwelling shortwave radiation at AWS02. (b) The result of adjusting the top-of-atmosphere (TOA) (back line) radiation with a constant (green line) and modified (cyan line) atmospheric transmissivity factor (see text) to best fit the measured clear sky daily average radiation (black dots).

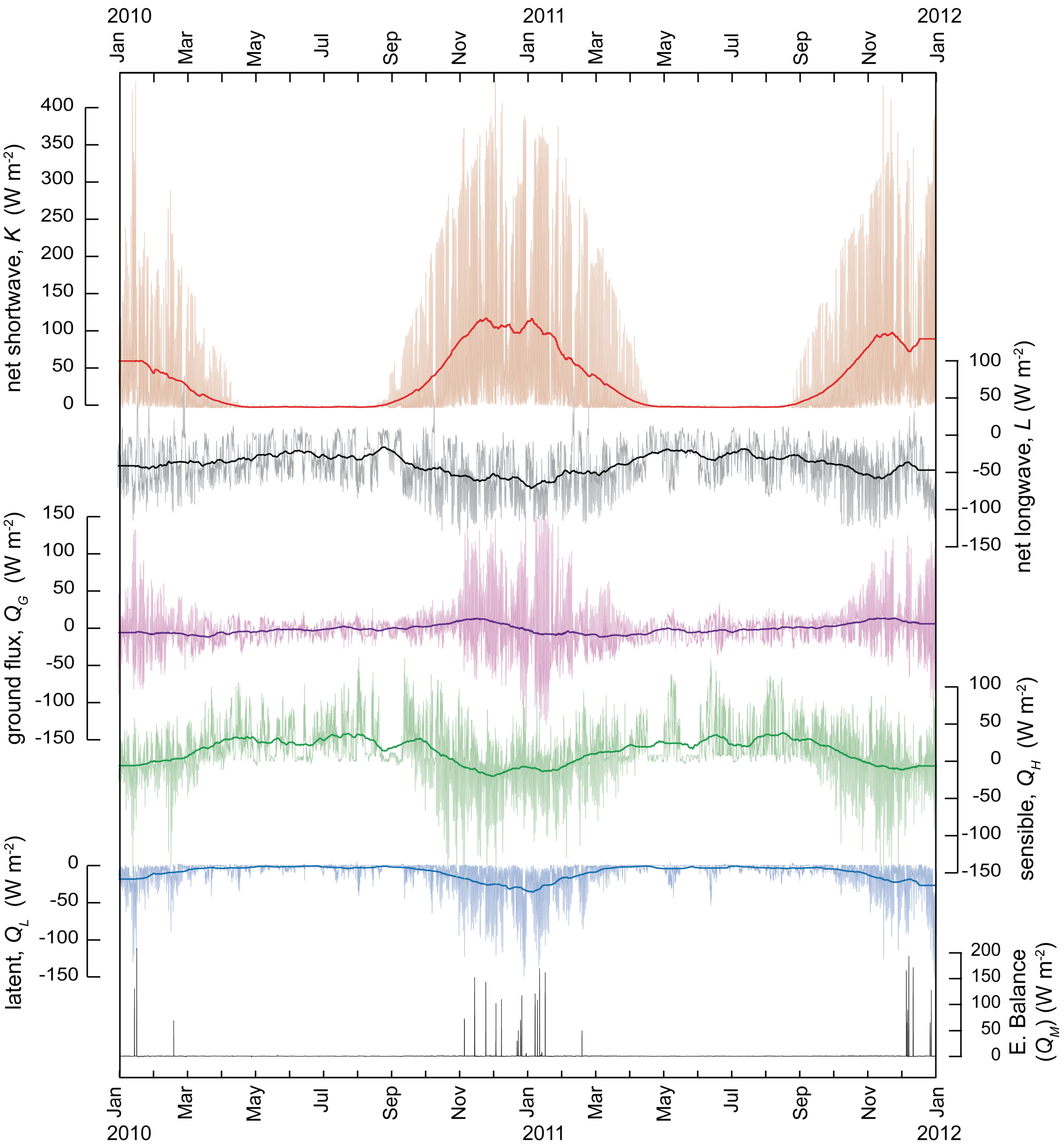

Supplementary Figure 7. Modeled energy balance components at AWS01 / L01 for modern conditions. Energy balance (bottom panel) is reached for almost all times without producing excess energy for melt. As modeled, melt accounts for ~0.5% of total ablation under modern conditions.

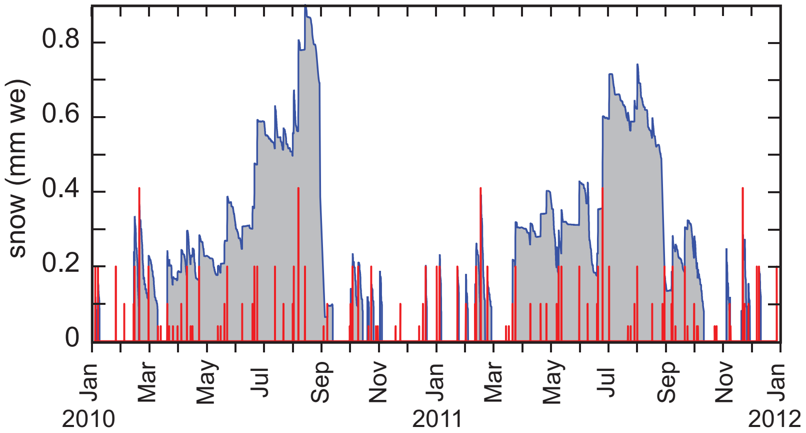

Supplementary Figure 8. Modeled snow pack evolution at AWS01/L01 for modern conditions. Red lines indicate snowfall events. Grey shaded area shows the buildup of a thin snow pack during winter and its rapid sublimation during the summer. Snow that falls in the summer sublimates almost immediately. Model results are consistent with the time-lapse camera snowfall record.

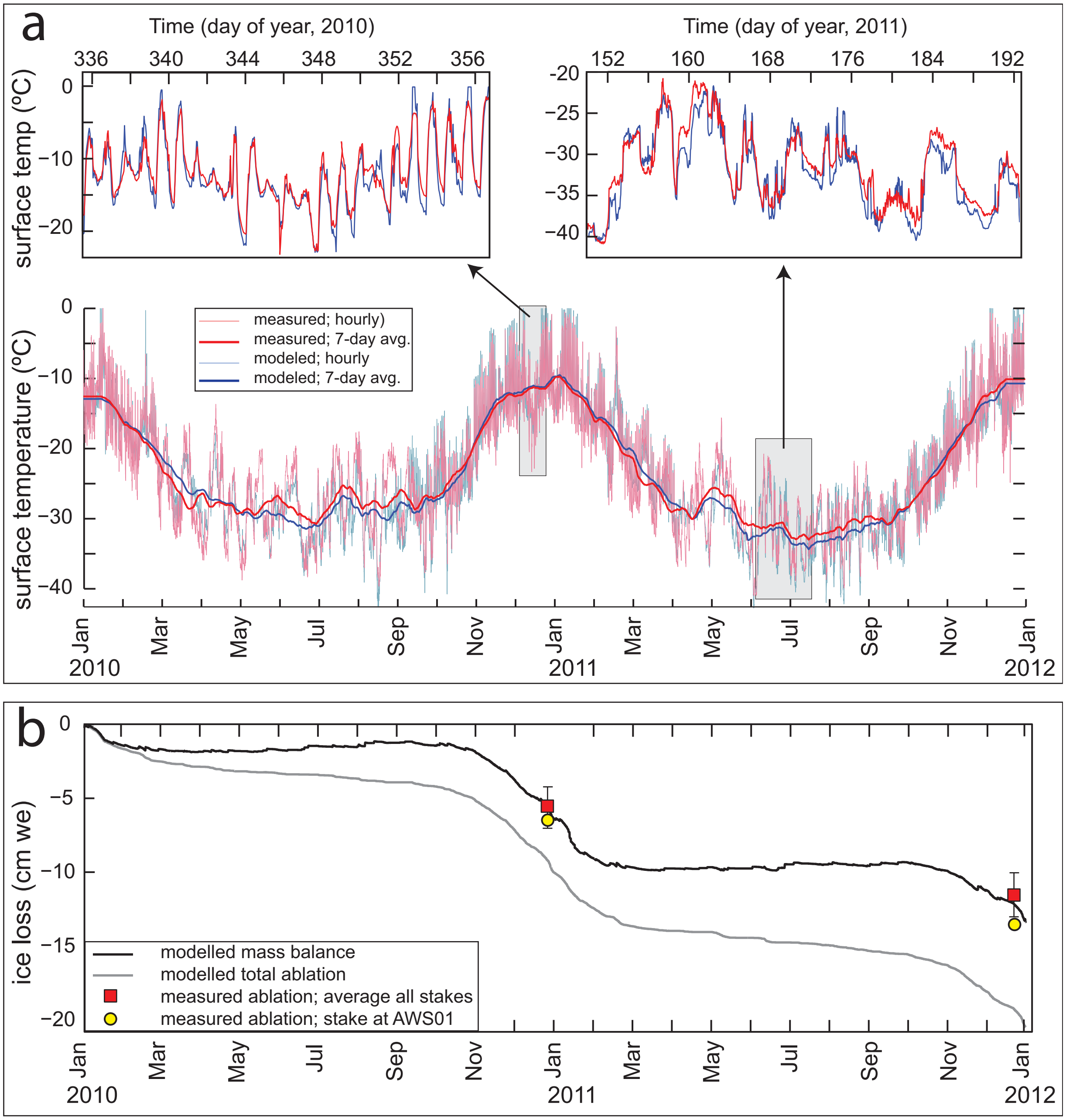

Supplementary Figure 9. Model validation at L01. (a) Comparison of measured (red lines) and modeled (blue lines) surface temperatures. Upper left and upper right panels show detailed views of the full time series, which is plotted below. The mean absolute error over the full time period is 1.4 °C. However, the MAE drops to 0.5 °C during summer months when peak ablation occurs. (b) Comparison of measured and modeled modern mass balance at AWS01. Measured and modeled ablation rates agree to within 1 cm a^{-1}. Also shown (grey line) is the modelled total ablation curve that would arise without the addition of snowfall.

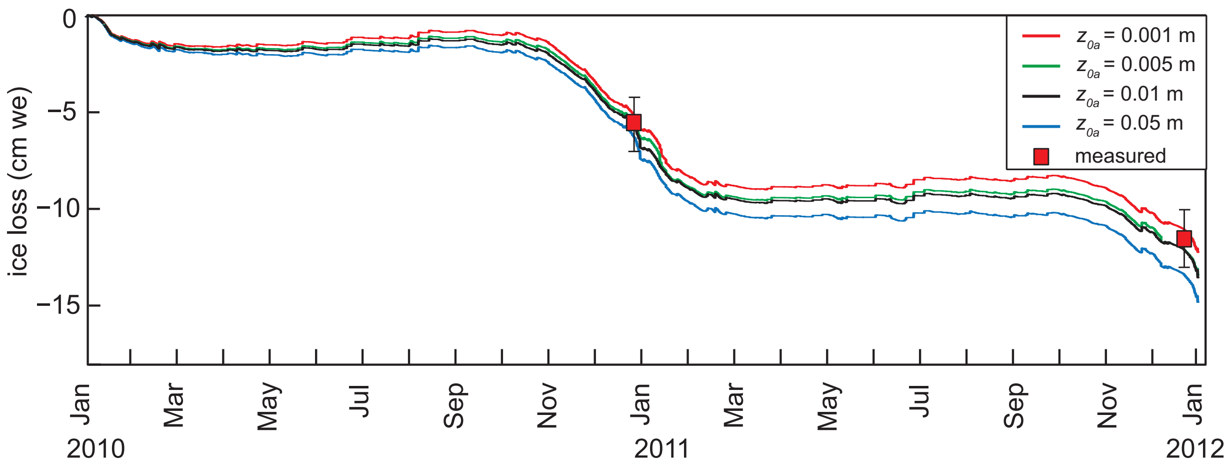

Supplementary Figure 10. Modeled ablation sensitivity to aerodynamic roughness (z_{0a}) evaluated over the validation data time period. The measured ablation (red squares and range bars) is the average of all stakes.

Supplementary References

1. Mackay SL, Marchant DR. Dating buried glacier ice using cosmogenic ^{3}He in surface clasts: theory and application to Mullins Glacier, Antarctica. *Quaternary Science Reviews* **140**, 75-100 (2016).

2. Mackay SL, Marchant DR, Lamp JL, Head JW. Cold-based debris-covered glaciers: Evaluating their potential as climate archives through studies of ground-penetrating radar and surface morphology. *Journal of Geophysical Research: Earth Surface* **119**, 2505-2540 (2014).
